# A Comparison of the Transcriptomes of Cowpeas in Response to Two Different Ionizing Radiations

**DOI:** 10.3390/plants10030567

**Published:** 2021-03-17

**Authors:** Ryulyi Kang, Eunju Seo, Aron Park, Woon Ji Kim, Byeong Hee Kang, Jeong-Hee Lee, Sang Hoon Kim, Si-Yong Kang, Bo-Keun Ha

**Affiliations:** 1 Department of Applied Plant Science, Chonnam National University, Gwangju 61186, Korea; fbfdl8556@gmail.com (R.K.); dmswn3562@gmail.com (E.S.); ironaron@naver.co (A.P.); dnswl007@naver.com (W.J.K.); rkdqudgml555@naver.com (B.H.K.); 2 BK21 FOUR Center for IT-Bio Convergence System Agriculture, Chonnam National University, Gwangju 61186, Korea; 3 Seeders Inc., Daejeon 34912, Korea; jhlee@seeders.co.kr; 4 Advanced Radiation Technology Institute, Korea Atomic Energy Research Institute, Jeongeup 56212, Korea; shkim80@kaeri.re.kr; 5 Department of Horticulture, College of Industrial Sciences, Kongju National University, Yesan 32439, Korea

**Keywords:** cowpea, gamma-ray, proton-beam, radio sensitivity, transcriptome, RNA-sequencing

## Abstract

In this study, gene expression changes in cowpea plants irradiated by two different types of radiation: proton-beams and gamma-rays were investigated. Seeds of the Okdang cultivar were exposed to 100, 200, and 300 Gy of gamma-rays and proton-beams. In transcriptome analysis, the 32, 75, and 69 differentially expressed genes (DEGs) at each dose of gamma-ray irradiation compared with that of the control were identified. A total of eight genes were commonly up-regulated for all gamma-ray doses. However, there were no down-regulated genes. In contrast, 168, 434, and 387 DEGs were identified for each dose of proton-beam irradiation compared with that of the control. A total of 61 DEGs were commonly up-regulated for all proton-beam doses. As a result of GO and KEGG analysis, the ranks of functional categories according to the number of DEGs were not the same in both treatments and were more diverse in terms of pathways in the proton-beam treatments than gamma-ray treatments. The number of genes related to defense, photosynthesis, reactive oxygen species (ROS), plant hormones, and transcription factors (TF) that were up-/down-regulated was higher in the proton beam treatment than that in gamma ray treatment. Proton-beam treatment had a distinct mutation spectrum and gene expression pattern compared to that of gamma-ray treatment. These results provide important information on the mechanism for gene regulation in response to two ionizing radiations in cowpeas.

## 1. Introduction

Ionizing radiation has been considered the most powerful source of mutagenesis for improving agricultural traits of various crops worldwide [1]. Since the 1960s, gamma-rays and X-rays have been commonly used to induce mutations during plant breeding [2]. In recent years, new mutant-derived cultivars have been developed using novel mutagens, such as cosmic rays and ion beams. These ionizing radiations have created various mutations that achieve high yield [3], early maturity [4], improvement of crop quality and nutritional traits [5], and resistance to biotic [6] and abiotic stress [7].

Gamma-rays are electromagnetic radiation with 0.2 keV μm^–1^ linear energy transfer (LET; the energy transferred per unit length), which can penetrate tissues [8]. The ion beams are composed of particles of various masses from protons to uranium atoms generated through particle accelerators and can have high-LET ranging from 22.5 keV μm^–1^ to 4000 keV μm^–1^ [8,9]. Compared with gamma-rays, ion beams show high relative biological effects (RBE), high mutation frequency, broad mutation spectrum, and induction of novel mutants [10].

Among ion beams, the proton-beams have recently attracted interest in mutation studies. Unlike heavy ion-beams, proton-beams have a lower LET (0.23–4.6 keV μm^−1^), but contain characteristic features of ion beams having a mass and an electrical charge [11]. Additionally, proton-beams have shown different mutant spectrums. In terms of characteristics of single nucleotide polymorphisms (SNPs), *Arabidopsis* irradiated with gamma-rays and carbon-ion beams [12], and *Glycine max* irradiated with gamma-rays [13] were had more transitions than transversions, whereas *Glycine max* irradiated with proton-beams showed the opposite result [14].

Plant gene expression can be changed by various stresses, such as ionizing radiation, UV-B, salinity, osmotic stress, and cold [15,16,17]. The N^+^ beam down-regulated the expression of genes involved in the photosynthetic pathway [18], and carbon-ion beams highly up-regulated the expression of genes involved in signal transduction mechanisms [19]. Therefore, comparative analysis of transcripts is very effective in the investigation of gene expression changes in response to various ionizing radiations. Recently, the emergence of next-generation sequencing (NGS)-based transcriptome analysis (RNA-seq) allows genome-wide identification of differentially expressed genes (DEGs) and provides a comprehensive overview of the metabolic pathways involved in stimulation through functional classification of the gene [20].

Cowpeas (*Vigna unguiculata* (L.) Walp.) are a versatile legume crop in which all stages of plant growth are used for human food and animal feed [21]. Recently, Lonardi et al. [22] sequenced the genome of cowpea IT97K-499-35 and identified 29,773 protein-coding loci with 12,514 alternatively spliced transcripts. Reference genome information made it possible to study the genome-wide gene expression responses to environmental stresses. Therefore, this study was conducted to investigate the transcriptional variations and the functions of the DEGs induced by proton-beams and gamma-rays in cowpeas.

## 2. Results

### 2.1. Transcriptional Variations Induced by Two Different Ionizing Radiations in Cowpeas

To compare transcriptional variations induced by gamma-rays and proton-beams in the cowpeas, RNA-sequencing analysis was performed with a total of 21 samples of control and irradiation treatments with three different doses (100, 200, and 300 Gy) for the two radiations. Each treatment was repeated independently three times. As a result of RNA-seq (Table S1), 600,059,830 transcriptome short reads (average length 101 bp) were collected from all samples. In the transcriptome short reads, bases with a phred score (Q) of less than 20, representing base quality, were trimmed, and reads with a trimmed read length of less than 25 bp were removed. The final value of the total length of trimmed reads/total length of raw reads was 80.00% using this preprocessing method. As a result of calculating the expression value by mapping 537,045,136 cleaned reads to 42,287 reference transcripts of cowpeas (Vunguiculata_469_v1.1), the mapping rate was approximately 91.46%. Additionally, among the 42,287 standard genes used for the analysis, 40,873 genes were expressed, and among them, 38,385 (93.91%) were genes with functional descriptions. These data indicated that reliable transcriptome data were available for subsequent differential analysis.

**Figure 1:**
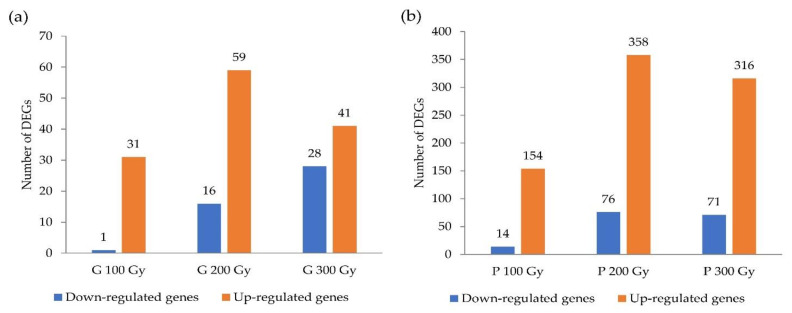
Number of differentially expressed genes (DEGs) that were identified with a comparison between the treatments and control in cowpeas. (**a**) Gamma-ray treatments, (**b**) Proton-beam treatments.

DEGs were screened between the treatments using log2FC (fold change) > 1 and p-adj (adjusted *p*-value) < 0.05 (Tables S2–S12). The value of log2FC greater than 1 was defined as upregulation, and less than 1 was defined as downregulation. The number of DEGs selected between the comparisons is shown in Figure 1. The 32, 75, and 69 DEGs at each dosage (100 Gy, 200 Gy, and 300 Gy) of gamma-ray irradiation compared with the control were identified. In contrast, 168, 434, and 387 DEGs were selected for each dose of proton-beam irradiation compared with the control. In the case of both sources, the number of DEGs was highest in the 200 Gy treatment, and slightly decreased in the 300 Gy treatment. Compared to that of gamma-rays, at all doses, the number of up-regulated and down- regulated DEGs were significantly higher in proton-beam treatments.

**Figure 2:**
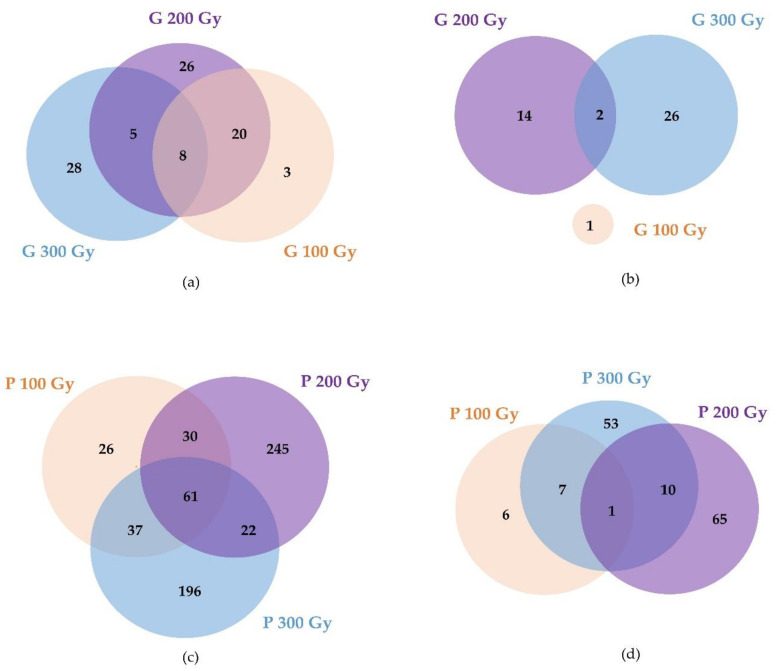
Venn diagram showing the number of common and specific differently expressed genes (DEGs) by gamma-ray (top) and proton-beam (bottom) treatments. (**a**,**c**) Up-regulated, (**b**,**d**) Down-regulated.

In the case of gamma-ray treatments (Figure 2a,b, Tables S8 and S9), 3, 26, and 28 DEGs were dose-specifically up-regulated in a dose-dependent manner for the 100 Gy, 200 Gy, and 300 Gy doses, and 1, 14, and 26 DEGs were down-regulated in a dose-dependent manner. Only eight genes were up-regulated in common at all doses, and these genes mainly encoded the Subtilisin-like serine endopeptidase family protein. In proton-beam treatments (Figure 2c,d, Tables S10 and S11), 26, 245, and 196 DEGs were up-regulated in a dose-dependent manner, and 6, 65, and 53 DEGs were down-regulated in a dose-dependent manner with 100 Gy, 200 Gy, and 300 Gy doses, respectively. A total of 61 DEGs were commonly up-regulated at all doses, and these genes encoded proteins, heat shock transcription factor A6B, the chaperone DnaJ-domain superfamily, NAC transcription factor-like 9, and beta glucosidase 15. One commonly down-regulated DEG encoded the nodulin MtN21/EamA-like transporter family protein. Overall, 133 and 759 DEGs were detected in gamma-ray treatments and proton-beam treatments, respectively. Among these, 95 overlapping DEGs were commonly detected in at least one or more doses for both radiations (Table S12). These DEGs included the GDSL-like Lipase/Acylhydrolase superfamily protein, NAD(P)-binding Rossmann-fold superfamily protein, lipoxygenase 1, and Subtilisin-like serine endopeptidase family protein.

There were no common DEGs in all doses of both sources. However, different DEGs encoding the same protein have been identified, and the definition of this protein was a subtilisin-like serine endopeptidase family protein. Interestingly, the DEGs encoding the heat shock protein 90.1, the heat shock transcription factor A6B, and the Chaperone DnaJ-domain superfamily protein, which increased in a dose-dependent manner in the proton-beam treatment, were not differentially expressed at all doses, but were differentially expressed only at 300 Gy in the gamma-ray treatments.

The qRT-PCR was performed to validate RNA-seq. A total of 15 genes were selected from the DEGs that commonly detected in gamma-ray treatments and proton-beam treatments (Figure S1). The relative expression levels of 15 genes obtained by qRT-PCR were similar to results of RNA-sequencing analysis.

### 2.2. Functional Categorization of DEGs Induced by Two Ionizing Radiations in Cowpeas

**Figure 3:**
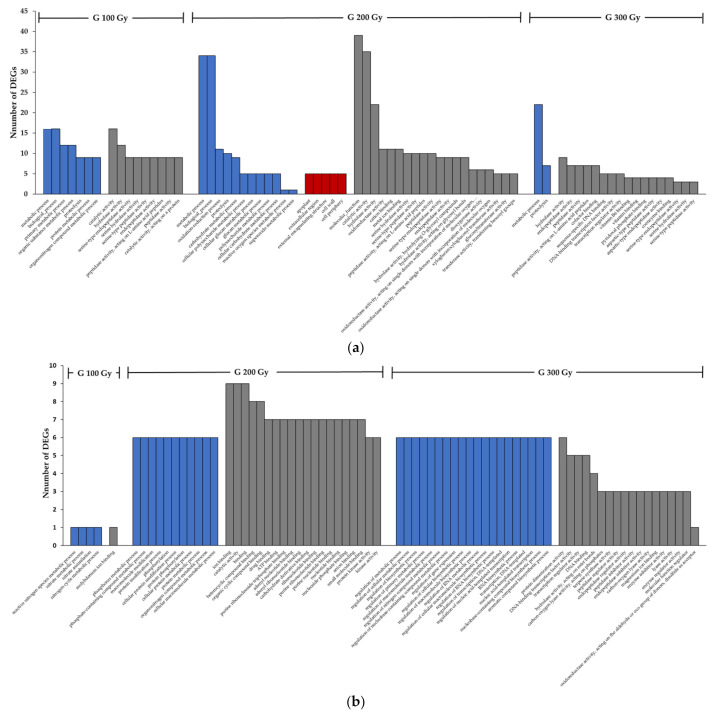
Gene Ontology (GO) term enrichment analysis of the up (**a**) down (**b**) regulated genes of cowpeas exposed to different dose rates of gamma-rays. (Blue bar: Biological Process, Red bar: Cellular Component, Grey bar: Molecular Function).

**Figure 4:**
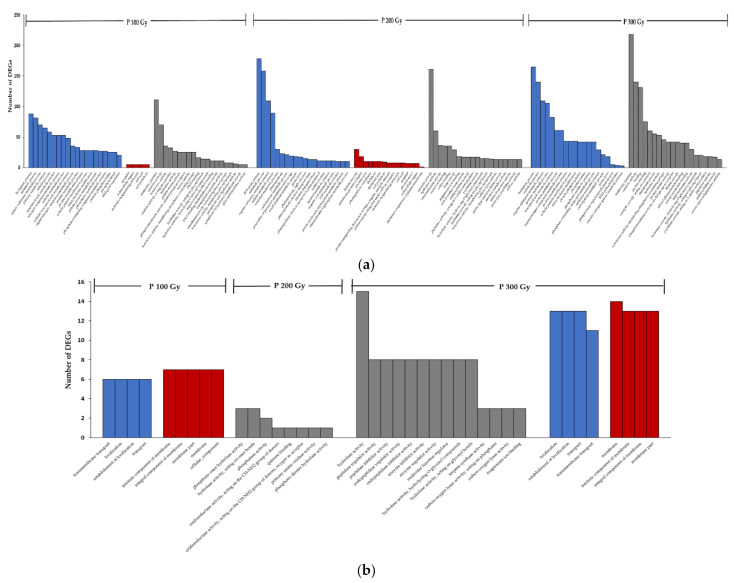
Gene Ontology (GO) term enrichment analysis of the up (**a**) down (**b**) regulated genes of cowpeas exposed to different dose rates of proton-beams. (Blue bar: Biological Process, Red bar: Cellular Component, Grey bar: Molecular Function).

To further understand the function of DEGs, Gene Ontology (GO) term enrichment analysis, and Kyoto Encyclopedia of Genes and Genomes (KEGG) enrichment were performed (Tables S2–S7). The GO term is presented in three independent categories Biological Process (BP), Molecular Function (MF), and Cellular Component (CC). Figure 3 and Figure 4 showed the top 20 enriched GO terms of the up/down-regulated genes by gamma-rays and proton-beams, respectively.

In a result of each treatment excluding overlapping up-regulated and down-regulated terms, 100 Gy, 200 Gy, and 300 Gy of the gamma-ray treatments showed 21, 81, and 60 terms, and the proton beam treatment showed 97, 157, and 79 terms. Overall, proton-beam treatments exhibited more diverse GO terms compared to gamma-ray treatments.

Comparing the GO results of up-regulated genes in the 200 Gy gamma-ray and proton-beam treatments, the most DEGs were detected in metabolic process and biological process terms in BP from both sources. However, differences existed in the next level. For gamma-ray treatments, oxidation-reduction process and proteolysis were the most enriched terms and for proton-beam treatments, organic substance metabolic process and cellular metabolic process were the most enriched terms. In CC, the apoplast the extracellular region were the most enriched terms in gamma-ray treatments, and membrane part and membrane protein complex were the most enriched terms in proton-beam treatments. In MF, catalytic activity, hydrolase activity, cation binding, metal ion binding, and oxidoreductase activity were similarly over-enriched in both sources, except for the molecular function term in gamma-ray treatments.

Table 1 and Table 2 show the enriched KEGG pathways of the up/down-regulated genes by gamma-ray and proton-beam. The DEGs were most involved in biosynthesis of other secondary metabolite pathways in all treatments. In proton-beam treatments, the next major pathways involved were environmental adaptation and signal transduction in 100 Gy, carbohydrate metabolism and energy metabolism in 200 Gy, environmental adaptation, folding, and sorting and degradation in 300 Gy. However, in the case of gamma-ray treatments, metabolism of terpenoids and polyketides in 100 Gy, lipid metabolism in 200 Gy, and environmental adaptation in 300 Gy were the prominent pathways. In particular, genes involved in environmental adaptation (plant-pathogen interaction, circadian rhythm—plant) pathway were generally up-regulated in all treatments of the proton beam treatment. However, in the gamma-ray treatment group, the number of up/down-regulated genes was the same or there were more down-regulated genes.

### 2.3. Clustering Analysis of DEGs Induced by Two Different Ionizing Radiations in Cowpeas

**Figure 5:**
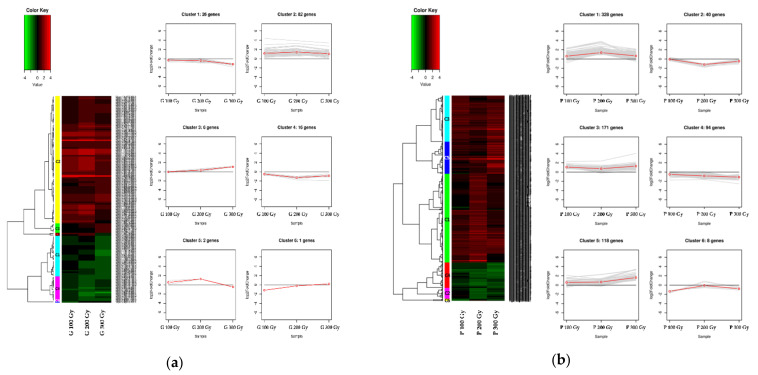
As a result of clustering analysis, heatmaps showing the level of differential expression of genes in each cluster. (**a**) Gamma-ray treatments; (**b**) proton-beam treatments. The line plot is a pattern representing the clusters expressed in the heatmaps. Up or down-regulated ranges from black to red (or green) with a fold-change scale bar shown above the heatmap.

A clustering analysis was performed to confirm the expression pattern of genes using differential expression gene information in 100 Gy vs. the control, 200 Gy vs. the control, and 300 Gy vs. the control comparisons for the two sources. The DEGs of the two sources were classified into six clusters each. In gamma-ray treatments (Figure 5a), the 82 of 133 DEGs belong to cluster 2 (C2). They showed a higher expression level than that of the control at all doses, and the level increased at 200 Gy and decreased at 300 Gy. In the GO analysis of these genes, most were distributed in biological process in BP, apoplast and extracellular region in CC, catalytic activity in MF. Additionally, KEGG analysis revealed that most of the genes were involved in biosynthesis of other secondary metabolites, lipid metabolism, and signal transduction. In proton-beam treatments (Figure 5b), 328 of 759 DEGs belonged to cluster 1(C1) and had a similar pattern to that of C2 of gamma-ray treatments. The GO analysis showed that most of the genes were distributed in the biological process in BP, membrane in CC, and catalytic activity in MF. Additionally, KEGG analysis revealed that most of the genes were involved in biosynthesis of other secondary metabolites, similar to that of gamma rays, followed by energy metabolism and carbohydrate metabolism. However, the proportion of genes involved in signal transduction and lipid metabolism was relatively small.

In contrast to the abovementioned clusters, C4 (16 DEGs) of the gamma-ray treatments showed lower expression levels than did the control at all doses, and these DEGs distributed in protein phosphorylation and phosphorylation in BP, and ion binding and catalytic activity in MF. Additionally, they were mainly involved in environmental adaptation, biosynthesis of other secondary metabolites and lipid metabolism pathway. C2 of proton-beam treatments showed a similar trend, including 40 DEGs. It also showed distribution in peptidase regulator activity, endopeptidase regulator activity, peptidase inhibitor activity, and endopeptidase inhibitor activity in MF. Furthermore, they were mainly involved in biosynthesis of other secondary metabolites, and metabolism of terpenoids and polyketides. The environmental adaptation pathway, which was most often observed in the C4 of gamma-ray treatments, was not seen in C2 of proton-beam treatments, but most in C3.

Additionally, the C5 (118 DEGs) of proton-beam treatments and C3 (six DEGs) of gamma-ray treatments had a pattern of increasing expression levels with dose, and the term oxidoreductase activity in MF was most commonly observed in both. However, the C4 (94 DEGs) of proton-beam treatments and the C1 (26 DEGs) of gamma-ray treatments, which showed a pattern of decreasing expression levels with dose, represented the term major hydrolase activity and protein dimerization activity in MF, respectively. Detailed information on the various clusters of each source is shown in Tables S13 and S14.

### 2.4. Target Gene Analysis of DEGs Induced by Two Different Ionizing Radiations in Cowpeas

To determine which signaling pathways were activated in response to the two ionizing radiations, we investigated the significant differences in the expression of genes related to ROS, plant hormones, defense signals, photosynthesis, and plant transcription factors (Table 3 and Table 4). For both sources, the largest number of DEGs were detected in plant hormones and transcription factors. For plant hormones, the genes related to SA and ABA were the most differentially expressed under both radiations. Interestingly, the AUX, CK, and BR genes were regulated only in proton-beam treatments, but not in gamma-ray treatments. Among them, CK-related DEGs were up-regulated at all doses, and none were down-regulated. For the TFs, 23 were regulated for both ionizing radiations. TALE was controlled only by gamma rays. bHLH, C3H, Dof, ERF, LBD, MIKC_MADS, and M-type_MADS were regulated by both sources. ARR-B, B3, bZIP, C2H2, CO-like, DBB, G2-like, GRF, HSF, MYB, MYB_related, NAC, Trihelix, WOX, and WRKY were regulated only by the proton beam.

The photosynthesis target genes were obtained only in 200 Gy of the proton-beam treatments. Most of these genes were matched to carbon fixation in photosynthetic organisms. However, both the ROS and defense genes were significantly up-regulated by 300 Gy in the proton beam treatments compared to that of gamma rays. The ROS-related genes included glutathione S-transferase TAU 8, 19, and peroxidase superfamily proteins. Furthermore, the defense-related genes included cation exchanger 1, cation exchanger 3, and β,-1,3-glucanase 1.

## 3. Discussion

Gamma rays have been commonly used as a mutagen to improve agricultural traits of crops [2]. Recently, it has been reported that proton-beam irradiation induced a different mutation spectrum compared to that of gamma-rays [14]. This means that the development of mutation breeding technology using proton-beams can lead to expansion of the genetic resource pool. Gamma-rays and ion beams caused different gene expression in plants that lead to changes in type, structure, and activity of proteins [23,24]. Therefore, transcriptome analysis can be a very useful approach to understanding physiological characteristics in response to stimuli. Several mutation breeding studies have been attempted to further understand the response to ionizing radiation through transcriptome analysis [24]. However, existing gene expression information is insufficient to fully understand the molecular mechanisms of gamma-rays and proton-beams. Therefore, this study was conducted to investigate the transcriptional variations and the functions of the DEGs induced by proton-beams and gamma-rays in cowpeas.

As a result of analyzing transcriptional variation in cowpeas irradiated with two ionizing radiations, there were clear differences in the numbers and types of DEGs. The numbers of DEGs were significantly higher with proton-beam treatments compared to that of gamma-rays. In both treatments, the largest number of DEGs were detected at 200 Gy, and the number of DEGs decreased at the next higher dose (300 Gy). The number of up-regulated DEGs was higher at all doses and sources than that of down-regulated DEGs. However, the opposite tendency was observed in rice irradiated with gamma rays and ion beams [25]. These DEGs were involved in a wide variety of biological functions such as plant development, abiotic stress responses, signaling, and plant secondary metabolism. Among them, genes encoding heat shock protein 90.1, the heat shock transcription factor A6B, and the Chaperone DnaJ-domain superfamily protein were dose-dependently expressed at all doses in the proton-beam treatments and differentially expressed only at 300 Gy of gamma-ray treatments. In rice, cosmic-ray and ion-beam treatments induced up-regulation of heat shock protein (HSP) genes, but gamma-ray treatments induced down-regulation of HSP genes [25]. The HSP and heat shock transcription factor were known to protect macromolecules to counteract stress, break down and recycle components of irreversibly damaged cells, and promote proper adaptive responses [26,27]. It was suggested that the misfolded proteins were generated or accumulated more in the proton-beam treatments than the gamma-ray treatments, and the degree increased in proportion to the dose. There were no common DEGs in all treatments of both sources. However, the different DEGs encoding the same protein have been identified, and the definition of this protein was a subtilisin-like serine endopeptidase family protein. This enzyme in *Arabidopsis thaliana* has been reported to be involved in the regulation of stomatal density and distribution [28] and auxin-induced lateral root formation [29]. Additionally, this gene was associated with stress-induced senescence in wheat [30].

The LET of an ion beam, such as a proton beam, is higher than that of a gamma ray, and this ion beam results in a higher mutation frequency and different mutation spectrum compared to that of gamma rays [11,31]. Furthermore, radiation with higher LET is known to cause more serious and more complex damage to DNA [32]. In the GO and KEGG analysis, the two treatments showed a different spectrum of terms and pathways, and a different number of related genes. In general, more terms and various pathways were revealed in the proton-beam treatments than the gamma-ray treatments. In other words, gene expression in cowpeas was more diversely and complexly regulated by a proton-beam with a higher LET than by gamma-rays, and the main mechanisms of each source were different. Additionally, it was complexly regulated at high doses (200 and 300 Gy) compared to low doses (100 Gy) in each source. Specifically, the DEGs involved in carbohydrate metabolism and energy metabolism pathways were excessively accumulated at 200 Gy and decreased at 300 Gy in the proton-beam treatments. However, the DEGs involved in environmental adaptation and folding, sorting, and degradation pathways were most prominent in all treatments (especially 300 Gy) of proton-beam compared to gamma-ray treatments. These results were similar to those of *Lemna minor* exposed to gamma rays, which changed the gene expression mechanism from acclimatization to a survival response by controlling energy distribution as the dose increased [33]. In the gamma-ray treatments, no corresponding trend was observed, which was considered a difference in crops.

As a result of confirming the expression pattern of DEGs through cluster analysis, most DEGs belonged to cluster 2 in the gamma-ray treatments. In proton-beam treatments, most of the DEGs belonged to cluster 1, which showed a similar pattern as did cluster 2 of gamma-rays. In both sources, the expression level of genes increased at 200 Gy and decreased at 300 Gy. This suggested that both sources may have a turning point in which gene expression tends to change with the dose of ionizing radiation. However, the function of the gene was not the same. The lipid metabolism (linoleic acid metabolism) pathway mainly observed in C2 of gamma-rays was observed in C5 of proton-beams. Additionally, many genes involved in energy metabolism (carbon fixation in photosynthetic organisms, photosynthesis) were observed in C1 of the proton beams but were not prominent in any clusters of gamma rays. This means that the response mechanism varies by controlling the expression of major genes according to the source and dose.

According to target gene analysis, the cowpeas stimulated by two ionizing radiations were most affected by plant hormones and TFs related genes. Hormones are involved in a wide range of biological processes and act as a defense in response to stress. ABA exhibits a prominent defense response against abiotic stresses, such as salinity and drought [34,35], and SA, JA, and ET are effective against biotic stresses, such as various pathogens and pests [36]. Additionally, AUX, GA, and CK are important hormones that promote plant growth [37]. In this study, ABA, SA, JA, ETH, and GA were regulated in gamma-ray treatments, and AUX, CK, and BR, including these were additionally regulated in the proton-beam treatments. In particular, the CK-related genes were only up-regulated in the proton-beam treatment. This hormone is known to be involved in cell division, leaf senescence, and nitrogen metabolism in plants [38]. The up-regulated cytokinin oxidase 5 is an enzyme that breaks down the hormone. The plant with increased cytokinin oxidase activity promoted root development and delayed shoot development [39]. When corn was treated to cold stress, the activity of this enzyme was increased at the root and shoot tips, and this enzyme was suggested to play a role in controlling the growth and development of organs [40]. Hormonal defense responses to adverse environmental conditions are known to rely on the complex crosstalk of signaling pathways rather than individual roles [35,37]. There is very little information on the role of hormones in responding to ionizing radiation. However, it is clear that compared to gamma-ray treatment, cowpea plants appear to require more complex hormonal mechanisms, including AUX, CK, and BR, to optimize development and growth by proton beam treatments.

TFs play an important role in the complex signaling processes, from the recognition of a stimulus to the expression of genes that respond to it [41]. These are classified according to the type of DNA-binding domains, and regulate metabolism by promoting or inhibiting specific gene expression in plants [42]. In this study, more diverse types of TFs were controlled by proton-beam treatments than by gamma-ray treatments. The Dof, ERF, and MADS were down-regulated in both sources, and most TFs tended to be up-regulated. In particular, bZIP, NAC, HSF, WRKY, and MYB were specifically up-regulated by the proton beams. However, these tended to be down-regulated overall by both ionizing radiation in rice irradiated with gamma-rays and carbon ion beams [25]. The AP2/EREBP was up-regulated by 800 Gy of gamma-rays, and C2H2 zinc finger and WRKY tended to be up-regulated at 100 Gy and down-regulated at 800 Gy in *Arabidopsis* plants [17]. In other words, plant metabolism is complexly regulated by varying the combination and concentration of TFs according to the ionizing radiation and the type of crop. WRKY controls the seed size, pathogen defense, senescence, and trichome development [43,44], and bZIP is involved in pathogen defense, light and stress signaling, seed maturation, and flower development [42]. DOF regulates metabolism in response to the environment, as well as tissue differentiation and seed development [45]. Additionally, gene expression regulation of TFs can ultimately lead to increased resistance to abiotic stress. The overexpression of the NAC gene significantly increased the resistance to drought and salt in rice without a growth delay [46]. Furthermore, ERF proteins, a subfamily of the APETALA2 (AP2)/ethylene-responsive-element-binding protein (EREBP), made tobacco plants resistant to drought and osmotic stress without having a significant effect on growth and development [47]. However, overexpressed DOF leads to growth defects [48]. Summarizing these results, it is proposed that TFs perform overlapping functions, such as plant development and stress tolerance, by controlling the combination and concentration in response to the two ionizing radiations and the specific role of individual TFs is not clear. However, it is clear that these TFs reacted more to proton-beams than gamma rays.

Notably, in the case of the photosynthesis target genes, they were all up-regulated only in 200 Gy proton-beam treatment. Most were involved in carbon fixation in photosynthetic organism’s pathway. This result was similar in that the low-dose gamma-rays increased the photosynthetic efficiency of *Arabidopsis thaliana*, and high-dose gamma-rays did not differ from those of the control group [49]. However, N+ -beam implantation induced biological damage through downregulation of photosynthesis-related genes in plants [50]. Our results indicated that gamma-ray and proton-beam irradiation in the cowpeas did not negatively affect the photosynthesis system. Rather, it implied that certain doses had the ability to generate more energy to respond or adapt to the stimulation of ionizing radiation.

Stresses, such as ionizing radiation, soil salinity, and drought, cause damage to plants, including oxidative stress caused by ROS, and affect plant physiology [51,52]. Plants activate defense mechanisms to protect themselves from this damage [52]. In ROS-related genes, glutathione S-transferase TAU and peroxidase superfamily proteins are induced by biotic and abiotic stress and play an important role in resistance to oxidative stress [53,54,55]. These were significantly up-regulated in the proton-beam treatments compared to the gamma-ray treatments. In other words, it is predicted that cowpeas were subjected to more oxidative stress by the proton-beam than by the gamma-ray. Similarly, the MDA and antioxidant enzyme activity of cowpeas irradiated with proton beam was higher than that of gamma rays [56]. The defense-related genes tend to be the same as the ROS-related genes. These include cation exchangers and calreticulin, which play a role in resistance to biotic and abiotic stress and plant protection against oxidative stress [57,58], and beta-1,3-glucanase, which retards fungal growth and reducing fruit spoilage [59].

When these results are summarized, it was concluded that the proton beam was a greater stressor than were gamma rays, which induces more disruption of normal protein production and accumulation of ROS. On the other hand, because many genes are involved in energy metabolism and environmental adaptation pathways were overexpressed in the proton-beam treatments, it is suggested that damage may have been minimized in terms of plant growth and development, and rather positive effects may have occurred. Additionally, a potential ability to induce positive changes in agricultural traits, such as flowering time, seed size, and resistance to biological and non-biological stress, can be proposed by the various genetic variations that respond to proton-beams.

## 4. Materials and Methods

### 4.1. Plant Materials and Radiation Treatments

A cowpea (*Vigna unguiculata* L. Walp) cultivar (Okdang) obtained from the Jeollanamdo Agricultural Research and Extension Services (JARES, Naju, Korea) was used in this study. The Okdang cultivar has an erect plant type with an intermediate plant habit and a high lodging resistance [60]. Okdang was irradiated with two different types of radiations, proton-beams and gamma-rays.

#### 4.1.1. Proton-Beam Irradiation

The seeds were irradiated with a 57 MeV proton-beam at 100 MeV using the proton linear accelerator at the Korea Multi-purpose Accelerator Complex (KOMAC) in Gyeongju, Korea. Seeds of the Okdang cultivar were exposed to three different doses of proton beams (100, 200, and 300 Gy). A total of 200 seeds were used for each treatment.

#### 4.1.2. Gamma-Ray Irradiation

Gamma-ray irradiation was conducted using the low-level irradiation facility containing ^60^CO as a source at the Korea Atomic Energy Research Institute. The irradiation doses were the same as the proton-beam irradiation. A total 200 seeds were used for each treatment.

The irradiated M1 seeds and non-irradiated control seeds were planted in individual cells of 50-cell plastic trays (27 × 53 × 11.2 cm) filled with potting mix (coco peat, peat moss, zeolite, pearlite, caldolomite, wetting agent, and fertilizer).

### 4.2. RNA Extraction

At 4 weeks after planting, young leaves from 21 plants were pooled for RNA extraction. Three independent pooled samples were prepared for each treatment. Isolation of total RNA was performed according to the manufacturer’s instructions for the TRIzol reagent (Invitrogen, Carlsbad, CA, USA). RNA was quantified based on absorbance at 260 nm measured with a Nanodrop 2000 spectrophotometer (Thermo Scientific, Waltham, MA, USA). After the extracted RNA was stained with Dyne LoadingSTAR (DYNEBIO Inc., Seongnam, Korea), the integrity of the sample was confirmed using 1.5% agarose gel electrophoresis.

### 4.3. cDNA Library Construction and Massively Parallel Sequencing

RNA-Seq paired end libraries were prepared using the Illumina TruSeq RNA Sample Preparation Kit v2 (catalog #RS-122-2001, Illumina, San Diego, CA, USA). With total RNA, mRNA was purified using poly (A) selection or rRNA depleted, then RNA was chemically fragmented and converted into single-stranded cDNA using random hexamer priming. Next, the second strand was generated to create double-stranded cDNA. A library was constructed with blunt-end cDNA fragments from ds-cDNA. Then, A-base was added to the blunt-end ready them for ligation of sequencing adapters. After the size selection of ligates, the ligated cDNA fragments that contained adapter sequences were enhanced via PCR using adapter specific primers. The library was quantified with a KAPA library quantification kit (KK4854, Kapa Biosystems, Cape Town, South Africa) following the manufacturer’s instructions. Each library was sequenced on the Illumina Hiseq2000 platform.

### 4.4. Preprocessing and Short Read Mapping

Sequence data, for which the quality of bp was indicated by Q ≥ 20, were extracted by SolexaQA [61]. Trimming resulted in reads with a mean length of 90.28 bp across all samples and a minimum length of 25 bp. Trimmed reads were mapped using the RNA-seq mapping algorithm implemented in bowtie2 (v2.1.0) software [62] to the reference transcripts of *Vigna unguiculata* (v1.1) downloaded from the Phytozome database (http://phytozome.jgi.doe.gov/ (accessed on 27 February 2021)), allowing all aligning with a maximum of two mismatches. The number of mapped clean reads for each gene was counted and then normalized with DESeq package in R [63] to avoid bias because of different sequencing amounts.

### 4.5. Identification of Differentially Expressed Genes (DEGs)

DEGs were identified by a ≥two-fold change in the number of mapped reads, a binomial test with a false discovery rate (FDR) ≤0.01, and a read count ≥ 100 between samples. The FDR was applied to identify the threshold *p*-value for multiple tests and was calculated using DESeq. Correlation analysis and hierarchical clustering was performed to group the genes according to patterns of expression using the AMAP library in R [64].

### 4.6. Functional Enrichment Analysis

To functionally annotate each DEG gene list, GO (Gene Ontology) analysis was conducted based on the sequence similarity (e-value cut off ≤ 1 × 10^−10^, best hits) of proteins in the Gene Ontology database [65]. The significance level was set to 0.05 using Blast2GO [66] and classified into functional categories: Biological Process (BP), Cellular Component (CC), and Molecular Function (MF). Additionally, the KEGG pathway was studied using the sequence similarity (e-value cut off ≤ 1 × 10^−10^, best hits) with the *Vigna radiata* protein in the KEGG database [67].

### 4.7. Target Gene Analysis

To obtain candidate genes related to ROS and hormones in cowpeas, protein sequences of *Arabidopsis* were used as a query, and 42,287 cowpea transcripts as a subject were compared and analyzed using the BLASTX (e-value ≤ 1 × 10^−10^, identity ≥ 50%). Repair system and photosynthesis-related genes were summarized by using the mung bean (*Vigna radiata*) gene information in the KEGG database. The comparative analysis used a BLAST tool and determined a gene aligned to the gene through a filter (e-value ≤ 1 × 10^−10^, identity ≥ 50%). The information of putative candidates of transcription factor (TFs) in cowpeas was obtained from PlantTFDB v3.0 [68].

### 4.8. Quantitative Reverse-Transcription PCR Validation of DEGs

For the validation analysis of mRNA expression levels quantitative real-time reverse transcription PCR (qRT-PCR) was conducted. The first-strand cDNA was synthesized from DNase-treated total RNA following the instructions of the SuperScript™ III First-Strand Synthesis SuperMix (Invitrogen, Carlsbad, CA, USA). Gene-specific primers were designed based on the nucleotide sequences of the selected DEGs for qRT-PCR analysis using Primer3 (v2.3.5) software (Table S15). The qRT-PCR were performed with a Bio-Rad iQ™ SYBR Green Supermix Kit (Invitrogen) using a StepOne Real-Time PCR System (Applied Biosystems, Foster City, CA, USA). The reaction mixtures (total volume of 20 μL) contained 100 ng of cDNA, each primer at 300 μM, 6 μL of ddH_2_O, and 10 μL of Bio-Rad iQ™ SYBR Green Supermix. The PCR conditions were set as follows: 95 °C for 3 min; 95 °C for 15 s, 60 °C for 60 s for 40 cycles; and the melt curve analysis was performed to confirm the absence of multiple products and the formation of primer dimers. The relative gene expression values were calculated by the 2^−△△Ct^ method [69]. The elongation factor 1β (EF-1β) gene was chosen as a reference gene for normalization of target gene expression in cowpeas [70]. For each of the three biological replicates, two technical replicates were included. For statistical analysis, the results were subjected to analysis of variance (ANOVA) using the SPSS version 25 software package (IBM, Armonk, NY, USA). Separation of means was performed using the Duncan’s multiple range test (DMRT) with significance at *p* < 0.05.

## 5. Conclusions

After irradiation of three different doses of gamma rays and proton beams to the cowpea seeds, transcriptional variations were investigated. The numbers of DEGs were significantly higher in the proton beams when compared to gamma ray treatments. These DEGs were involved in a wide variety of biological functions, such as plant development, abiotic stress responses, signaling, and plant secondary metabolism. However, there were no common DEGs in all treatments for both sources. As a result of the GO and KEGG analysis, the two treatments showed a different spectrum of terms and pathways, and a difference in the number related genes. In other words, the gene expressions of cowpeas in response to the two ionizing radiations were highly diverse and complex, and their specific mechanisms were different. These results provided insights into genes that were regulated by gamma rays and proton beams and their functional relationships.

## Figures and Tables

**Table 1:** Kyoto Encyclopedia of Genes and Genomes (KEGG) enrichment analysis of the up- and down-regulated genes of cowpeas exposed to different dose rates of gamma-rays.

Major Classification	Sub Classification	Number of DEGs	
		Up-Regulated	Down-Regulated
G 100 Gy			
Metabolism	Amino acid metabolism	1	0
Metabolism	Biosynthesis of other secondary metabolites	19	0
Metabolism	Carbohydrate metabolism	3	0
Metabolism	Energy metabolism	0	1
Metabolism	Lipid metabolism	3	0
Metabolism	Metabolism of terpenoids and polyketides	4	0
G 200 Gy			
Cellular Processes	Transport and catabolism	1	0
Environmental Information Processing	Membrane transport	0	1
Environmental Information Processing	Signal transduction	6	0
Metabolism	Amino acid metabolism	4	0
Metabolism	Biosynthesis of other secondary metabolites	30	5
Metabolism	Carbohydrate metabolism	5	0
Metabolism	Glycan biosynthesis and metabolism	0	1
Metabolism	Lipid metabolism	8	4
Metabolism	Metabolism of terpenoids and polyketides	5	1
Metabolism	Overview	3	2
Organismal Systems	Environmental adaptation	0	6
G 300 Gy			
Environmental Information Processing	Signal transduction	1	2
Metabolism	Amino acid metabolism	4	1
Metabolism	Biosynthesis of other secondary metabolites	21	14
Metabolism	Carbohydrate metabolism	0	2
Metabolism	Energy metabolism	1	0
Metabolism	Glycan biosynthesis and metabolism	0	1
Metabolism	Metabolism of terpenoids and polyketides	2	3
Metabolism	Overview	4	0
Organismal Systems	Environmental adaptation	3	3

**Table 2:** Kyoto Encyclopedia of Genes and Genomes (KEGG) enrichment analysis of the up- and down-regulated genes in cowpeas exposed to different dose rates of proton-beams.

Major Classification	Sub Classification	Number of DEGs	
		Up-Regulated	Down-Regulated
P 100 Gy			
Cellular Processes	Transport and catabolism	1	0
Environmental Information Processing	Membrane transport	1	0
Environmental Information Processing	Signal transduction	14	0
Genetic Information Processing	Folding, sorting and degradation	6	0
Genetic Information Processing	Transcription	2	0
Metabolism	Amino acid metabolism	4	3
Metabolism	Biosynthesis of other secondary metabolites	48	7
Metabolism	Carbohydrate metabolism	10	0
Metabolism	Energy metabolism	4	0
Metabolism	Lipid metabolism	6	1
Metabolism	Metabolism of cofactors and vitamins	1	3
Metabolism	Metabolism of other amino acids	9	1
Metabolism	Metabolism of terpenoids and polyketides	5	0
Metabolism	Overview	3	0
Organismal Systems	Environmental adaptation	40	0
P 200 Gy			
Cellular Processes	Transport and catabolism	11	0
Environmental Information Processing	Membrane transport	5	0
Environmental Information Processing	Signal transduction	19	5
Genetic Information Processing	Folding, sorting and degradation	8	0
Genetic Information Processing	Transcription	1	0
Metabolism	Amino acid metabolism	16	4
Metabolism	Biosynthesis of other secondary metabolites	149	41
Metabolism	Carbohydrate metabolism	68	13
Metabolism	Energy metabolism	64	0
Metabolism	Glycan biosynthesis and metabolism	0	1
Metabolism	Lipid metabolism	6	1
Metabolism	Metabolism of cofactors and vitamins	6	0
Metabolism	Metabolism of other amino acids	14	1
Metabolism	Metabolism of terpenoids and polyketides	13	7
Metabolism	Nucleotide metabolism	1	2
Metabolism	Overview	49	0
Organismal Systems	Environmental adaptation	29	4
P 300 Gy			
Cellular Processes	Transport and catabolism	6	0
Environmental Information Processing	Membrane transport	7	0
Environmental Information Processing	Signal transduction	15	1
Genetic Information Processing	Folding, sorting and degradation	31	0
Genetic Information Processing	Transcription	2	0
Genetic Information Processing	Translation	8	0
Human Diseases	Endocrine and metabolic diseases	0	1
Metabolism	Amino acid metabolism	13	3
Metabolism	Biosynthesis of other secondary metabolites	103	11
Metabolism	Carbohydrate metabolism	20	4
Metabolism	Energy metabolism	4	1
Metabolism	Lipid metabolism	24	4
Metabolism	Metabolism of cofactors and vitamins	1	3
Metabolism	Metabolism of other amino acids	17	1
Metabolism	Metabolism of terpenoids and polyketides	9	0
Metabolism	Overview	6	2
Organismal Systems	Environmental adaptation	79	13

**Table 3:** The differently expressed genes (DEGs) list as related to defense, photosynthesis, and ROS of cowpeas exposed to different dose rates of gamma-rays and proton-beams.

Category		Gene ID	E-Value	Identity	ID	Log2 Fold Change						Arabi-Defline *
						G 100 Gy	G 200 Gy	G 300 Gy	P 100 Gy	P 200 Gy	P 300 Gy	
Defense	calcium transporters	Vigun05g102200	7 × 10^−165^	73.02	AT3G51860	−0.40	0.13	0.24	0.97	1.10	0.93	cation exchanger 3
	calcium transporters	Vigun06g076000	1 × 10^−135^	64.37	AT3G51860	1.00	0.85	1.07	0.56	1.09	1.24	cation exchanger 1
	calcium transporters	Vigun08g119600	3 × 10^−130^	70.66	AT3G51860	0.97	0.84	1.04	0.26	0.99	1.18	cation exchanger 1
	chitinases	Vigun11g168800	8 × 10^−85^	53.46	AT2G43590	−0.60	−0.68	−0.90	−0.27	−1.10	−1.13	homolog of carrot EP3-3 chitinase
	ER-folding	Vigun05g013700	0	66.12	AT5G28540	0.03	0.22	0.15	0.39	0.45	1.10	heat shock cognate protein 70-1
	ER-folding	Vigun05g054000	0	75.64	AT1G08450	0.00	0.33	0.37	0.75	0.68	1.06	calreticulin 3
	ER-folding	Vigun06g184800	0	56.63	AT5G42020	0.31	0.35	0.03	0.85	0.62	1.19	heat shock protein 70B
	MAPK cascade linked to PAMP defense/innate immunity	Vigun05g118000	8 × 10^−17^	56.14	AT4G23550	0.23	0.32	0.77	−0.02	1.07	0.64	WRKY DNA-binding protein 49
	CALRETICULIN 3	Vigun05g054000	0	75.64	AT1G08450	0.00	0.33	0.37	0.75	0.68	1.06	calreticulin 3
	PR2	Vigun01g111100	1 × 10^−126^	54.47	AT3G57260	1.01	1.49	0.98	0.22	0.56	1.47	beta-1,3-glucanase 1
	PR2	Vigun01g111300	4 × 10^−102^	56.49	AT3G57260	1.24	1.70	1.29	0.31	0.61	1.57	beta-1,3-glucanase 1
	PR2	Vigun11g037800	4 × 10^−103^	52.38	AT3G57260	−0.30	−0.70	−0.45	−0.34	−0.57	−1.34	Glycosyl hydrolase superfamily protein
	PRXCA	Vigun06g141200	4 × 10^−111^	54.06	AT3G49110	0.19	0.77	0.30	0.36	0.20	1.54	Peroxidase superfamily protein
Photosynthesis	Photosynthesis	Vigun01g147100	7 × 10^−94^	93.62	vra:106758006	−0.32	−0.05	0.79	0.29	2.42	0.70	photosystem II reaction center PSB28 protein
	Photosynthesis	Vigun11g069900	2 × 10^−96^	70.93	vra:106761818	0.52	0.75	0.46	0.14	1.19	0.66	photosystem II reaction center protein A
	Photosynthesis—antenna proteins	Vigun01g226400	1 × 10^−159^	93.55	vra:106758023	−0.25	0.13	1.10	0.68	2.46	1.21	light harvesting complex photosystem II
	Photosynthesis—antenna proteins	Vigun09g238500	2 × 10^−157^	96.59	vra:106761217	−0.05	0.19	0.30	0.18	1.08	0.20	photosystem I light harvesting complex gene 5
	Carbon fixation in photosynthetic organisms	Vigun02g098200	0	99.24	vra:106777824	−0.11	0.07	0.14	0.45	1.03	0.29	phosphoribulokinase
	Carbon fixation in photosynthetic organisms	Vigun04g097100	4 × 10^−116^	86.26	vra:106773825	−0.06	0.04	0.28	0.37	1.25	0.43	Ribulose bisphosphate carboxylase (small chain) family protein
	Carbon fixation in photosynthetic organisms	Vigun07g217500	3 × 10^−113^	50.74	vra:106754509	−0.29	0.03	0.17	0.43	1.10	0.32	Inositol monophosphatase family protein
	Carbon fixation in photosynthetic organisms	Vigun07g291200	0	97.37	vra:106772090	0.04	0.30	0.21	0.23	0.96	0.40	Transketolase
	Carbon fixation in photosynthetic organisms	Vigun11g181300	3 × 10^−134^	57.67	vra:106757953	−0.02	0.05	0.31	0.12	1.13	0.21	fructose-bisphosphate aldolase 2
	Carbon fixation in photosynthetic organisms	VigunL056600	3 × 10^−100^	87.35	vra:106779100	0.21	0.47	0.34	0.05	1.13	0.69	ribulose-bisphosphate carboxylases
ROS	ROS breakdown	Vigun01g050200	3 × 10^−94^	62.96	AT1G17180	0.01	0.13	0.19	0.81	0.53	1.28	glutathione S-transferase TAU 19
	ROS breakdown	Vigun01g050300	7 × 10^−96^	60.38	AT1G17180	−0.01	0.15	−0.10	0.89	0.49	1.36	glutathione S-transferase TAU 19
	ROS breakdown	Vigun01g058300	1 × 10^−93^	63.43	AT1G17180	0.01	0.13	0.19	0.81	0.53	1.29	glutathione S-transferase TAU 19
	ROS breakdown	Vigun05g112200	6 × 10^−69^	52.68	AT3G09270	0.09	0.55	0.27	0.68	0.65	1.22	glutathione S-transferase TAU 8
	ROS breakdown	Vigun05g177100	2 × 10^−149^	57.67	AT1G17020	0.48	1.00	0.79	0.07	0.28	1.13	senescence-related gene 1
	ROS induced genes	Vigun01g102600	0	53.37	AT5G48570	0.15	0.59	0.89	0.48	1.50	0.77	FKBP-type peptidyl-prolyl cis-trans isomerase family protein
	ROS induced genes	Vigun06g052000	2 × 10^−117^	54.28	AT5G55050	0.29	0.52	0.77	0.74	0.86	1.54	GDSL-like Lipase/Acylhydrolase superfamily protein
	ROS induced genes	Vigun11g168800	1 × 10^−68^	52.61	AT2G43570	−0.60	−0.68	−0.90	−0.27	−1.10	−1.13	homolog of carrot EP3-3 chitinase
	ROS induced genes, ROS transport to apoplast	Vigun06g224100	1 × 10^−127^	58.65	AT5G64120	3.01	3.30	2.72	−0.64	1.79	1.98	Peroxidase superfamily protein
	ROS production	Vigun06g141200	2 × 10^−110^	54.06	AT3G49110	0.19	0.77	0.30	0.36	0.20	1.54	Peroxidase superfamily protein
	ROS production	Vigun07g235300	4 × 10^−78^	63.69	AT2G26400	−0.16	0.02	0.22	0.72	1.16	0.70	RmlC-like cupins superfamily protein
	ROS production	Vigun09g130300	6 × 10^−175^	77.59	AT5G05340	0.19	0.58	0.32	0.32	0.51	1.24	Peroxidase superfamily protein
	ROS production	Vigun09g139800	4 × 10^−160^	58.33	AT5G24530	0.27	0.34	0.92	1.11	0.97	1.01	2-oxoglutarate (2OG) and Fe(II)-dependent oxygenase superfamily protein
	ROS production	Vigun10g149500	0	61.51	AT1G01190	1.60	1.52	1.18	−0.12	0.55	0.88	cytochrome P450, family 78, subfamily A, polypeptide 6

* Arabi-defline: *Arabidopsis thaliana* gene’s description. The box color indicates the gene expression level from green (down-regulated) to white to red (up-regulated). The black lined box indicates a significant difference.

**Table 4:** The differently expressed genes (DEGs) list related to plant hormone, TF of cowpeas exposed to different dose rates of gamma-rays and proton-beams.

Category		Gene ID	E-Value	Identity	ID	Log2 Fold Change						Arabi-Defline *
						G 100 Gy	G 200 Gy	G 300 Gy	P 100 Gy	P 200 Gy	P 300 Gy	
Plant hormone	JA	Vigun05g272300	0	56.17	AT1G55020	1.44	2.01	0.79	0.53	0.70	2.01	lipoxygenase 1
	JA	Vigun09g243000	0	64.68	AT5G42650	0.08	0.36	0.18	0.52	0.32	1.06	allene oxide synthase
	JA	Vigun10g168900	0	57.01	AT1G55020	0.57	0.83	0.05	0.00	−0.25	1.27	lipoxygenase 1
	JA	Vigun11g024700	5 × 10^−131^	51.83	AT2G06050	0.47	0.46	0.24	0.99	0.27	1.05	12-oxophytodienoate reductase 2
	JA	Vigun11g025000	5 × 10^−127^	50.52	AT2G06050	0.42	0.47	0.33	1.00	0.26	1.12	12-oxophytodienoate reductase 2
	JA	Vigun09g060800	2 × 10^−14^	53.06	AT4G28910	−0.15	−0.34	−0.45	−0.83	−0.87	−1.04	ABI five binding protein 3
	SA	Vigun01g111100	3 × 10^−126^	54.47	AT3G57260	1.01	1.49	0.98	0.22	0.56	1.47	beta-1,3-glucanase 1
	SA	Vigun01g111300	1 × 10^−101^	56.49	AT3G57260	1.24	1.70	1.29	0.31	0.61	1.57	beta-1,3-glucanase 1
	SA	Vigun06g125400	1 × 10^−16^	55.56	AT4G17500	−0.65	−0.88	−1.02	−0.32	−1.00	−1.36	Integrase-type DNA-binding superfamily protein
	SA	Vigun09g066100	9 × 10^−12^	53.42	AT4G17500	−0.19	−0.29	−0.96	−0.24	−1.43	−1.40	Integrase-type DNA-binding superfamily protein
	SA	Vigun10g147000	1 × 10^−16^	51.47	AT4G17500	−0.13	−0.40	−0.58	−0.39	−0.50	−1.27	Integrase-type DNA-binding superfamily protein
	SA	Vigun11g037800	1 × 10^−102^	52.38	AT3G57260	−0.30	−0.70	−0.45	−0.34	−0.57	−1.34	Glycosyl hydrolase superfamily protein
	SA	Vigun05g118000	5 × 10^−21^	59.65	AT3G01080	0.23	0.32	0.77	−0.02	1.07	0.64	WRKY DNA-binding protein 49
	SA	Vigun06g122600	2 × 10^−14^	51.72	AT3G01080	0.36	0.20	0.36	1.15	0.78	0.75	WRKY DNA-binding protein 30
	SA	Vigun08g112200	1 × 10^−22^	55.07	AT2G40750	0.25	−0.04	0.53	1.46	1.35	0.67	WRKY DNA-binding protein 70
	SA	Vigun03g272400	3 × 10^−100^	55.98	AT2G23620	−0.26	−0.52	−0.96	−0.27	−1.11	−1.08	methyl esterase 1
	ETH	Vigun03g006800	8 × 10^−152^	73.4	AT1G05010	0.11	0.43	0.29	0.37	0.66	1.20	ethylene-forming enzyme
	ETH	Vigun06g125400	1 × 10^−18^	54.29	AT2G44840	−0.65	−0.88	−1.02	−0.32	−1.00	−1.36	Integrase-type DNA-binding superfamily protein
	ETH	Vigun10g147000	9 × 10^−20^	52	AT2G44840	−0.13	−0.40	−0.58	−0.39	−0.50	−1.27	Integrase-type DNA-binding superfamily protein
	AUX	Vigun02g197800	4 × 10^−161^	60.91	AT4G27070	0.50	0.57	0.18	0.53	−0.18	1.19	Pyridoxal-5\'-phosphate-dependent enzyme family protein
	AUX	Vigun02g197900	2 × 10^−166^	60.14	AT5G54810	0.45	0.66	0.05	0.52	−0.20	1.19	Pyridoxal-5\'-phosphate-dependent enzyme family protein
	AUX	Vigun02g200300	5 × 10^−167^	62.79	AT5G54810	0.45	0.68	0.04	0.53	−0.18	1.21	Pyridoxal-5\'-phosphate-dependent enzyme family protein
	AUX	Vigun08g204500	0	75.13	AT5G55250	−0.31	−0.25	−0.77	−0.19	−1.08	−0.47	IAA carboxylmethyltransferase 1
	AUX	Vigun01g186200	1 × 10^−121^	51.34	AT5G62000	−0.08	−0.11	−0.39	−0.45	−1.19	−0.77	auxin response factor 2
	AUX	Vigun09g055300	3 × 10^−89^	62.02	AT5G57090	−0.35	−0.86	−0.83	−0.58	−1.29	−1.67	Auxin efflux carrier family protein
	CK	Vigun01g223000	4 × 10^−141^	50.44	AT5G05870	0.30	0.74	0.67	1.27	1.16	1.17	UDP-Glycosyltransferase superfamily protein
	CK	Vigun09g248900	0	68.08	AT1G75450	0.07	0.19	0.42	1.41	1.15	0.49	cytokinin oxidase 5
	CK	Vigun05g236200	1 × 10^−15^	54.55	AT3G16857	−0.10	0.12	0.79	0.63	1.30	0.59	Homeodomain-like superfamily protein
	BR	Vigun04g063600	0	74	AT3G30180	−0.13	−0.33	−0.98	−0.98	−1.36	−0.84	brassinosteroid-6-oxidase 2
	BR	Vigun10g199600	0	58.4	AT2G07050	−0.04	−0.39	0.58	0.46	1.20	0.06	Terpenoid cyclases family protein
	ABA	Vigun01g111100	4 × 10^−126^	54.47	AT3G57260	1.01	1.49	0.98	0.22	0.56	1.47	beta-1,3-glucanase 1
	ABA	Vigun01g111300	1 × 10^−101^	56.49	AT3G57260	1.24	1.70	1.29	0.31	0.61	1.57	beta-1,3-glucanase 1
	ABA	Vigun02g108000	0	60.09	AT2G29090	0.32	0.42	−0.19	1.36	1.52	−0.19	cytochrome P450, family 707, subfamily A, polypeptide 1
	ABA	Vigun11g037800	1 × 10^−102^	52.38	AT3G57260	−0.30	−0.70	−0.45	−0.34	−0.57	−1.34	Glycosyl hydrolase superfamily protein
	ABA	Vigun11g124400	1 × 10^−63^	54.36	AT1G52340	2.33	2.85	1.92	−0.30	1.23	2.07	NAD(P)-binding Rossmann-fold superfamily protein
	ABA	Vigun05g118000	2 × 10^−19^	56.14	AT1G80840	0.23	0.32	0.77	−0.02	1.07	0.64	WRKY DNA-binding protein 49
	ABA	Vigun06g125400	3 × 10^−14^	67.86	AT2G40220	−0.65	−0.88	−1.02	−0.32	−1.00	−1.36	Integrase-type DNA-binding superfamily protein
	ABA	Vigun08g103000	5 × 10^−164^	71.24	AT3G50500	−0.24	−0.62	−0.36	−0.13	−0.59	−1.03	Protein kinase superfamily protein
	ABA	Vigun09g066100	1 × 10^−10^	59.65	AT2G40220	−0.19	−0.29	−0.96	−0.24	−1.43	−1.40	Integrase-type DNA-binding superfamily protein
	ABA	Vigun10g147000	1 × 10^−14^	64.29	AT2G40220	−0.13	−0.40	−0.58	−0.39	−0.50	−1.27	Integrase-type DNA-binding superfamily protein
	ABA	Vigun01g182800	0	66.91	AT5G06530	−0.13	−0.11	0.52	0.73	1.42	0.86	ABC-2 type transporter family protein
	ABA	Vigun07g020000	0	78.09	AT5G06530	0.07	0.42	0.80	−0.45	1.19	0.45	ABC-2 type transporter family protein
	ABA	Vigun07g208100	0	61.09	AT1G71960	−0.49	−1.16	−0.86	−0.53	−0.99	−0.53	ATP-binding cassette family G25
	GA	Vigun01g203100	2 × 10^−30^	54.39	AT1G09530	−0.20	−0.80	−1.10	0.19	−0.92	−0.80	phytochrome interacting factor 3
	GA	Vigun11g176000	1 × 10^−16^	50	AT1G09530	0.03	0.37	0.59	0.67	1.16	0.38	cryptochrome-interacting basic-helix-loop-helix 1
TF	ARR-B	Vigun05g236200	2 × 10^−15^	54.24	Vun001281	−0.10	0.12	0.79	0.63	1.30	0.59	Homeodomain-like superfamily protein
	B3	Vigun01g186200	5 × 10^−85^	65.37	Vun008946	−0.08	−0.11	−0.39	−0.45	−1.19	−0.77	auxin response factor 2
	bHLH	Vigun01g203100	3 × 10^−22^	67.21	Vun004469	−0.20	−0.80	−1.10	0.19	−0.92	−0.80	phytochrome interacting factor 3
	bHLH	Vigun02g030100	1 × 10^−136^	100	Vun010029	−0.07	−0.03	0.69	0.45	1.28	0.39	basic helix-loop-helix (bHLH) DNA-binding superfamily protein
	bHLH	Vigun11g176000	0	99.73	Vun001364	0.03	0.37	0.59	0.67	1.16	0.38	cryptochrome-interacting basic-helix-loop-helix 1
	bZIP	Vigun05g188500	3 × 10^−22^	51.55	Vun006014	0.60	0.64	0.77	0.99	1.17	0.15	Basic-leucine zipper (bZIP) transcription factor family protein
	bZIP	Vigun05g211100	1 × 10^−22^	52.58	Vun006014	0.20	0.45	0.10	1.05	1.14	0.39	Basic-leucine zipper (bZIP) transcription factor family protein
	C2H2	Vigun05g249900	6 × 10^−164^	91.67	Vun007540	0.09	−0.01	0.52	0.56	1.08	0.23	C2H2-like zinc finger protein
	C2H2	Vigun07g117400	3 × 10^−111^	55.04	Vun000818	−0.12	−0.26	−0.36	−0.44	−0.87	−1.23	Indeterminate (ID)-domain 2
	C3H	Vigun04g004600	2 × 10^−61^	66.93	Vun007793	−0.50	−1.16	−1.14	−0.19	−1.23	−0.92	CCCH-type zinc finger family protein
	CO-like	Vigun03g017700	2 × 10^−11^	66.67	Vun007845	0.19	0.32	0.69	0.19	1.61	0.24	B-box type zinc finger protein with CCT domain
	DBB	Vigun03g017700	4 × 10^−11^	56	Vun005093	0.19	0.32	0.69	0.19	1.61	0.24	B-box type zinc finger protein with CCT domain
	Dof	Vigun03g336800	9 × 10^−27^	64.71	Vun009020	−0.18	−0.66	−0.58	−0.38	−0.67	−1.00	cycling DOF factor 3
	Dof	Vigun05g041000	2 × 10^−26^	70.69	Vun010069	−0.09	−0.68	−1.02	−0.90	−1.10	−1.07	cycling DOF factor 2
	ERF	Vigun06g125400	3 × 10^−28^	68.92	Vun008975	−0.65	−0.88	−1.02	−0.32	−1.00	−1.36	Integrase-type DNA-binding superfamily protein
	ERF	Vigun09g066100	2 × 10^−22^	60	Vun004651	−0.19	−0.29	−0.96	−0.24	−1.43	−1.40	Integrase-type DNA-binding superfamily protein
	ERF	Vigun10g147000	5 × 10^−27^	66.22	Vun004557	−0.13	−0.40	−0.58	−0.39	−0.50	−1.27	Integrase-type DNA-binding superfamily protein
	G2-like	Vigun05g236200	1 × 10^−68^	58.91	Vun009285	−0.10	0.12	0.79	0.63	1.30	0.59	Homeodomain-like superfamily protein
	GRF	Vigun01g171000	7 × 10^−132^	99.46	Vun004052	0.03	0.10	−0.44	−0.26	−1.24	−0.62	growth-regulating factor 4
	HSF	Vigun01g137100	1 × 10^−38^	63.83	Vun002051	−0.04	0.14	0.70	1.01	1.32	2.36	heat shock transcription factor A6B
	HSF	Vigun09g224500	6 × 10^−36^	60.64	Vun002051	−0.26	−0.05	1.24	2.28	2.38	4.01	heat shock transcription factor A2
	LBD	Vigun02g150500	1 × 10^−125^	99.45	Vun010679	0.61	0.75	1.11	0.19	0.88	0.24	LOB domain-containing protein 37
	LBD	Vigun03g285600	9 × 10^−168^	99.56	Vun009383	0.39	0.72	1.11	−0.11	1.09	−0.09	LOB domain-containing protein 39
	MIKC_MADS	Vigun02g125600	6 × 10^−19^	50.68	Vun005031	−0.19	−0.82	−1.06	0.01	−0.89	−1.09	K-box region and MADS-box transcription factor family protein
	MIKC_MADS	Vigun03g126100	1 × 10^−104^	97.44	Vun010515	−0.47	−0.58	−1.04	0.11	−0.64	−0.45	K-box region and MADS-box transcription factor family protein
	M-type_MADS	Vigun02g125600	1 × 10^−19^	54.93	Vun006638	−0.19	−0.82	−1.06	0.01	−0.89	−1.09	K-box region and MADS-box transcription factor family protein
	M-type_MADS	Vigun03g126100	3 × 10^−46^	59.02	Vun006638	−0.47	−0.58	−1.04	0.11	−0.64	−0.45	K-box region and MADS-box transcription factor family protein
	MYB	Vigun02g147400	0	100	Vun007952	0.25	0.58	0.43	0.62	1.21	−0.24	myb domain protein 73
	MYB	Vigun03g281700	0	100	Vun007814	0.16	0.39	0.30	0.87	1.08	0.10	myb domain protein 73
	MYB_related	Vigun02g147400	6 × 10^−20^	51.47	Vun001892	0.25	0.58	0.43	0.62	1.21	−0.24	myb domain protein 73
	MYB_related	Vigun03g281700	2 × 10^−21^	50	Vun001892	0.16	0.39	0.30	0.87	1.08	0.10	myb domain protein 73
	MYB_related	Vigun06g096400	4 × 10^−74^	60.14	Vun002263	−0.31	−0.91	−0.93	−0.67	−1.02	−0.90	Homeodomain-like superfamily protein
	MYB_related	Vigun07g078900	1 × 10^−173^	100	Vun008084	−0.10	−0.32	−0.29	−0.53	−0.37	−1.04	Homeodomain-like superfamily protein
	MYB_related	Vigun08g140800	2 × 10^−175^	97.15	Vun002263	−0.21	−0.83	−0.65	−0.63	−0.80	−1.26	Homeodomain-like superfamily protein
	NAC	Vigun10g154100	5 × 10^−54^	63.49	Vun011756	−0.04	0.52	0.52	1.53	0.99	1.82	
	NAC	Vigun10g154300	8 × 10^−52^	63.49	Vun011756	0.10	0.49	0.83	1.46	1.11	1.82	NAC transcription factor-like 9
	NAC	Vigun10g154700	8 × 10^−47^	59.2	Vun011756	0.14	0.51	0.86	1.48	1.16	1.86	NAC transcription factor-like 9
	NAC	VigunL060400	7 × 10^−55^	62.4	Vun011756	−0.04	0.46	0.60	1.44	0.94	1.72	NAC transcription factor-like 9
	NAC	VigunL060000	5 × 10^−52^	63.49	Vun011756	0.11	0.50	0.83	1.47	1.12	1.82	NAC transcription factor-like 9
	TALE	Vigun08g034600	6 × 10^−41^	81.37	Vun011787	4.38	3.90	3.40	−0.41	1.81	2.63	KNOTTED-like from Arabidopsis thaliana
	Trihelix	Vigun04g131800	4 × 10^−102^	91.11	Vun009396	0.40	0.09	0.27	0.67	1.19	0.54	Duplicated homeodomain-like superfamily protein
	WOX	Vigun03g004000	5 × 10^−27^	72.31	Vun003771	−0.37	−0.48	−0.46	−0.55	−0.90	−1.04	WUSCHEL related homeobox 1
	WRKY	Vigun05g118000	4 × 10^−23^	64.91	Vun007741	0.23	0.32	0.77	−0.02	1.07	0.64	WRKY DNA-binding protein 49
	WRKY	Vigun06g122600	0	100	Vun001400	0.36	0.20	0.36	1.15	0.78	0.75	WRKY DNA-binding protein 30
	WRKY	Vigun08g112200	4 × 10^−27^	64.79	Vun005168	0.25	−0.04	0.53	1.46	1.35	0.67	WRKY DNA-binding protein 70

* Arabi-defline: *Arabidopsis thaliana* gene’s description. The box color indicates the gene expression level from green (down-regulated) to white to red (up-regulated). The black line of box mean significant difference.

## Data Availability

All data are contained within the article.

## Supplementary Materials

The following are available online at https://www.mdpi.com/2223-7747/10/3/567/s1, Figure S1: Quantitative Reverse-Transcription PCR validation of differently expressed genes (DEGs). Blue and orange bar graph represent relative expression level from RNA-seq and qRT-PCR, respectively. Error bars represent the SE for three independent replicates, Table S1: Read statistics for RNA sequencing of cowpeas exposed to gamma-rays and proton beams, Table S2: The information and functional analysis results of differentially expressed genes (DEGs) in cowpeas exposed to 100 Gy gamma-ray, Table S3: The information and functional analysis results of differentially expressed genes (DEGs) in cowpeas exposed to 200 Gy gamma-ray, Table S4: The information and functional analysis results of differentially expressed genes (DEGs) in cowpeas exposed to 300 Gy gamma-ray, Table S5: The information and functional analysis results of differentially expressed genes (DEGs) in cowpeas exposed to 100 Gy proton-beam, Table S6: The information and functional analysis results of differentially expressed genes (DEGs) in cowpeas exposed to 200 Gy proton-beam, Table S7: The information and functional analysis results of differentially expressed genes (DEGs) in cowpeas exposed to 300 Gy proton-beam, Table S8: The differently expressed genes (DEGs) list for each area of Venn diagram created using up-regulated DEGs in gamma-ray treatments, Table S9: The differently expressed genes (DEGs) list for each area of Venn diagram created using down-regulated DEGs in gamma-ray treatments, Table S10: The differently expressed genes (DEGs) list for each area of Venn diagram created using up-regulated DEGs in proton-beam treatments, Table S11: The differently expressed genes (DEGs) list for each area of Venn diagram created using down-regulated DEGs in proton-beam treatments Table S12: The differently expressed genes (DEGs) list for each area of Venn diagram created using DEGs in gamma-ray and proton-beam treatments, Table S13: The comprehensive results of clustering analysis performed using differently expressed genes (DEGs) in gamma-ray treatments, Table S14: The comprehensive results of clustering analysis performed using differently expressed genes (DEGs) in proton-beam treatments, Table S15: Primers used for qRT-PCR analysis for validation of differently expressed genes (DEGs).

## References

[1] Chen W., Li H., Shi L., Bai H.T. (2016). Sensitivity of Lavender to Proton, Electron, and Gamma Radiation. Korean J. Hortic Sci. Technol..

[2] Kodym A., Afza R. (2003). Physical and chemical mutagenesis. Plant Functional Genomics.

[3] Balooch A.W., Soomro A.M., Naqvi M.H., Bughio H.R., Bughio M.S. (2006). Sustainable enhancement of rice (*Oryza sativa* L.) production through the use of mutation breeding. Plant. Mutat. Rep..

[4] Ismachin M. (2006). A significant contribution of mutation techniques to rice breeding in Indonesia. Plant. Mutat. Rep..

[5] Ceballos H., Sanchez T., Denyer K., Tofino A.P., Rosero E.A., Dufour D., Smith A., Morante N., Perez J.C., Fahy B. (2008). Induction and identification of a small-granule, high-amylose mutant in cassava (*Manihot esculenta* Crantz). J. Agric. Food Chem..

[6] Peiris R., Wickramasinghe T., Indrasena S. (2008). M 127-A promising tomato variety developed through induced mutation tech-nique. Induced Plant Mutations in the Genomics Era, Proceedings of the International Joint FAO/IAEA Symposium, Rome, Italy, 3–5 June 2008.

[7] Do K.T., Dao M.S., Hung P.Q., Nguyen T.C. (2006). Rice mutation improvement for short duration, high yield and tolerance to adverse conditions in Mekong Delta of Viet Nam. Plant. Mutat. Rep..

[8] Oladosu Y., Rafii M.Y., Abdullah N., Hussin G., Ramli A., Rahim H.A., Miah G., Usman M. (2016). Principle and application of plant mutagenesis in crop improvement: A review. Biotechnol. Biotechnol. Equip..

[9] Ryuto H., Fukunishi N., Hayashi Y., Ichida H., Abe T., Kase M., Yano Y. (2008). Heavy-ion beam irradiation facility for biological samples in RIKEN. Plant Biotechnol..

[10] Tanaka A., Shikazono N., Hase Y. (2010). Studies on Biological Effects of Ion Beams on Lethality, Molecular Nature of Mutation, Mutation Rate, and Spectrum of Mutation Phenotype for Mutation Breeding in Higher Plants. J. Radiat. Res..

[11] Lee Y.-M., Jo Y.D., Lee H.-J., Kim Y.-S., Kim D.S., Kim J.-B., Kang S.-Y., Kim S.H. (2015). DNA damage and oxidative stress induced by proton beam in Cymbidium hybrid. Hortic. Environ. Biotechnol..

[12] Yoshihara R., Hase Y., Sato R., Takimoto K., Narumi I. (2010). Mutational effects of different LET radiations in rpsL transgenic Arabidopsis. Int. J. Radiat. Biol..

[13] Lee K.J., Kim D.S., Kim J.-B., Jo S.-H., Kang S.-Y., Choi H.-I., Ha B.-K. (2016). Identification of candidate genes for an early-maturing soybean mutant by genome resequencing analysis. Mol. Genet. Genom..

[14] Kim W.J., Ryu J., Im J., Kim S.H., Kang S.-Y., Lee J.-H., Jo S.-H., Ha B.-K. (2018). Molecular characterization of proton beam-induced mutations in soybean using genotyping-by-sequencing. Mol. Genet. Genom..

[15] Yoon M.Y., Kim M.Y., Shim S., Kim K.D., Ha J., Shin J.H., Kang S., Lee S.-H. (2016). Transcriptomic Profiling of Soybean in Response to High-Intensity UV-B Irradiation Reveals Stress Defense Signaling. Front. Plant Sci..

[16] Kreps J.A., Wu Y., Chang H.-S., Zhu T., Wang X., Harper J.F. (2002). Transcriptome Changes for Arabidopsis in Response to Salt, Osmotic, and Cold Stress. Plant Physiol..

[17] Hwang S.-G., Kim D.S., Kim J.-B., Hwang J.E., Park H.M., Jang C.S. (2016). Transcriptome analysis of reproductive-stage Arabidopsis plants exposed gamma-ray irradiation at various doses. Int. J. Radiat. Biol..

[18] Chen Q., Ya H., Wang W., Jiao Z. (2014). RNA-seq reveals the downregulated proteins related to photosynthesis in growth-inhibited rice seedlings induced by low-energy N+ beam implantation. Genet. Mol. Res..

[19] Rakwal R., Kimura S., Shibato J., Nojima K., Kim Y.-K., Nahm B.H., Jwa N.-S., Endo S., Tanaka K., Iwahashi H. (2008). Growth retardation and death of rice plants irradiated with carbon ion beams is preceded by very early dose- and time-dependent gene expression changes. Mol. Cells.

[20] Shankar R., Bhattacharjee A., Jain M. (2016). Transcriptome analysis in different rice cultivars provides novel insights into desiccation and salinity stress responses. Sci. Rep..

[21] Gómez C. (2004). Cowpea: Post-Harvest Operations.

[22] Lonardi S., Muñoz-Amatriaín M., Liang Q., Shu S., Wanamaker S.I., Lo S., Tanskanen J., Schulman A.H., Zhu T., Luo M. (2019). The genome of cowpea (*Vigna unguiculata* [L.] Walp.). Plant J..

[23] Kim S.-H., Song M.R., Lee K.J., Hwang S.-G., Jang C.S., Kim J.-B., Kim S.H., Ha B.-K., Kang S.-Y., Kim D.-S. (2012). Genome-wide transcriptome profiling of ROS scavenging and signal transduction pathways in rice (*Oryza sativa* L.) in response to different types of ionizing radiation. Mol. Biol. Rep..

[24] Ya H., Chen Q., Wang W., Chen W., Qin G., Jiao Z. (2012). Gene expression profiles in promoted-growth rice seedlings that germinated from the seeds implanted by low-energy N+ beam. J. Radiat. Res..

[25] Hwang J.E., Hwang S.-G., Kim S.-H., Lee K.J., Jang C.S., Kim J.-B., Kim S.H., Ha B.-K., Ahn J.-W., Kang S.-Y. (2013). Transcriptome profiling in response to different types of ionizing radiation and identification of multiple radio marker genes in rice. Physiol. Plant..

[26] Ohama N., Sato H., Shinozaki K., Yamaguchi-Shinozaki K. (2017). Transcriptional Regulatory Network of Plant Heat Stress Response. Trends Plant Sci..

[27] Hahn J.-S., Hu Z., Thiele D.J., Iyer V.R. (2004). Genome-Wide Analysis of the Biology of Stress Responses through Heat Shock Transcription Factor. Mol. Cell. Biol..

[28] Berger D., Altmann T. (2000). A subtilisin-like serine protease involved in the regulation of stomatal density and distribution in Arabidopsis thaliana. Genome Res..

[29] Neuteboom L.W., Ng J.M., Kuyper M., Clijdesdale O.R., Hooykaas P.J., Van Der Zaal B.J. (1999). Isolation and characterization of cDNA clones corresponding with mRNAs that accumulate during auxin-induced lateral root formation. Plant Mol. Biol..

[30] Roberts I.N., Caputo C., Kade M., Criado M.V., Barneix A.J. (2011). Subtilisin-like serine proteases involved in N remobilization during grain filling in wheat. Acta Physiol. Plant..

[31] Okamura M., Yasuno N., Ohtsuka M., Tanaka A., Shikazono N., Hase Y. (2003). Wide variety of flower-color and –shape mutants regenerated from leaf cultures irradiated with ion beams. Nucl. Instrum. Meth. Phys. Res..

[32] Sage E., Harrison L. (2011). Clustered DNA lesion repair in eukaryotes: Relevance to mutagenesis and cell survival. Mutat. Res. Mol. Mech. Mutagen..

[33] Van Hoeck A., Horemans N., Nauts R., Van Hees M., Vandenhove H., Blust R. (2017). Lemna minor plants chronically exposed to ionising radiation: RNA-seq analysis indicates a dose rate dependent shift from acclimation to survival strategies. Plant Sci..

[34] Zhang J., Schurr U., Davies W.J. (1987). Control of Stomatal Behaviour by Abscisic Acid which Apparently Originates in the Roots. J. Exp. Bot..

[35] Peleg Z., Blumwald E. (2011). Hormone balance and abiotic stress tolerance in crop plants. Curr. Opin. Plant Biol..

[36] Bari R., Jones J.D.G. (2009). Role of plant hormones in plant defence responses. Plant Mol. Biol..

[37] Verma V., Ravindran P., Kumar P.P. (2016). Plant hormone-mediated regulation of stress responses. BMC Plant Biol..

[38] Argueso C.T., Ferreira F.J., Kieber J.J. (2009). Environmental perception avenues: The interaction of cytokinin and environmental response pathways. Plant. Cell Environ..

[39] Werner T., Motyka V., Strnad M., Schmülling T. (2001). Regulation of plant growth by cytokinin. Proc. Natl. Acad. Sci. USA.

[40] Brugière N., Jiao S., Hantke S., Zinselmeier C., Roessler J.A., Niu X., Jones R.J., Habben J.E. (2003). Cytokinin Oxidase Gene Expression in Maize Is Localized to the Vasculature, and Is Induced by Cytokinins, Abscisic Acid, and Abiotic Stress. Plant Physiol..

[41] Hussain S.S., Kayani M.A., Amjad M. (2011). Transcription factors as tools to engineer enhanced drought stress tolerance in plants. Biotechnol. Prog..

[42] Jakoby M., Weisshaar B., Dröge-Laser W., Vicente-Carbajosa J., Tiedemann J., Kroj T., Parcy F. (2002). bZIP transcription factors in Arabidopsis. Trends Plant Sci..

[43] Luo M., Dennis E.S., Berger F., Peacock W.J., Chaudhury A. (2005). MINISEED3 (MINI3), a WRKY family gene, and HAIKU2 (IKU2), a leucine-rich repeat (LRR) KINASE gene, are regulators of seed size in Arabidopsis. Proc. Natl. Acad. Sci. USA.

[44] Eulgem T., Rushton P.J., Robatzek S., Somssich I.E. (2000). The WRKY superfamily of plant transcription factors. Trends Plant Sci..

[45] Noguero M., Atif R.M., Ochatt S., Thompson R.D. (2013). The role of the DNA-binding One Zinc Finger (DOF) transcription factor family in plants. Plant Sci..

[46] Zheng X., Chen B., Lu G., Han B. (2009). Overexpression of a NAC transcription factor enhances rice drought and salt tolerance. Biochem. Biophys. Res. Commun..

[47] Trujillo L., Menendez C., Ochogavia M.E., Hernandez I., Borras O., Rodriguez R., Coll Y., Arrieta J.G., Banguela A., Ramirez R. (2009). Engineering drought and salt tolerance in plants using SodERF3, a novel sugarcane ethylene responsive factor. Biotechnol. Appl..

[48] Kang H.-G., Singh K.B. (2000). Characterization of salicylic acid-responsive, Arabidopsis Dof domain proteins: Overexpression of OBP3 leads to growth defects. Plant J..

[49] Vanhoudt N., Horemans N., Wannijn J., Nauts R., Van Hees M., Vandenhove H. (2014). Primary stress responses in Arabidopsis thaliana exposed to gamma radiation. J. Environ. Radioact..

[50] Ya H., Chen Q., Wang W., Cheng Y. (2014). Gene expression characteristics of growth-inhibited rice seedlings induced by low-energy N+-beam implantation. Genet. Mol. Res..

[51] Kim D.S., Kim J.-B., Goh E.J., Kim W.-J., Kim S.H., Seo Y.W., Jang C.S., Kang S.-Y. (2011). Antioxidant response of Arabidopsis plants to gamma irradiation: Genome-wide expression profiling of the ROS scavenging and signal transduction pathways. J. Plant Physiol..

[52] Das K., Choudhury A.R. (2014). Reactive Oxygen Species (ROS) and responses of antioxidants as ROS-scavengers during environmental stress in plants. Front. Environ. Sci..

[53] Sharma R., Sahoo A., Devendran R., Jain M. (2014). Over-expression of a rice tau class glutathione s-transferase gene improves tolerance to salinity and oxidative stresses in Arabidopsis. PLoS ONE.

[54] Sarowar S., Kim E.N., Kim Y.J., Ok S.H., Kim K.D., Hwang B.K., Shin J.S. (2005). Overexpression of a pepper ascorbate peroxidase-like 1 gene in tobacco plants enhances tolerance to oxidative stress and pathogens. Plant Sci..

[55] Pandey V.P., Awasthi M., Singh S., Tiwari S., Dwivedi U.N. (2017). A comprehensive review on function and application of plant peroxidases. Biochem. Anal. Biochem..

[56] Kang R., Seo E., Kim G., Park A., Kim W.J., Kang S.-Y., Ha B.-K. (2020). Radio Sensitivity of Cowpea Plants after Gamma-Ray and Proton-Beam Irradiation. Plant Breed. Biotechnol..

[57] Baliardini C., Corso M., Verbruggen N. (2016). Transcriptomic analysis supports the role of CATION EXCHANGER 1 in cellular homeostasis and oxidative stress limitation during cadmium stress. Plant Signal. Behav..

[58] Joshi R., Paul M., Kumar A., Pandey D. (2019). Role of calreticulin in biotic and abiotic stress signalling and tolerance mechanisms in plants. Gene.

[59] Xu X., Tian S. (2008). Salicylic acid alleviated pathogen-induced oxidative stress in harvested sweet cherry fruit. Postharvest Biol. Technol..

[60] Kim D.-K., Choi J.-G., Kwon O.-D., Lee K.-D., Ryu A.K.-I. (2018). Cowpea Cultivar, ’Okdang’, with an Intermediate Plant Habit and Erect Plant Type. Korean J. Breed. Sci..

[61] Cox M.P., Peterson D.A., Biggs P.J. (2010). SolexaQA: At-a-glance quality assessment of Illumina second-generation sequencing data. BMC Bioinform..

[62] Langmead B., Trapnell C., Pop M., Salzberg S.L. (2009). Ultrafast and memory-efficient alignment of short DNA sequences to the human genome. Genome Biol..

[63] Anders S., Huber W. (2010). Differential expression analysis for sequence count data. Genome Biol..

[64] Lucas A. Amap: Another Multidimensional Analysis Package; R Package Version 0.8-14. https://rdrr.io/cran/amap/.

[65] Ashburner M., Ball C.A., Blake J.A., Botstein D., Butler H., Cherry J.M., Davis A.P., Dolinski K., Dwight S.S., Eppig J.T. (2000). Gene Ontology: Tool for the unification of biology. Nat. Genet..

[66] Conesa A., Götz S., García-Gómez J.M., Terol J., Talón M., Robles M. (2005). Blast2GO: A universal tool for annotation, visualization and analysis in functional genomics research. Bioinformatics.

[67] Ogata H., Goto S., Sato K., Fujibuchi W., Bono H., Kanehisa M. (1999). KEGG: Kyoto Encyclopedia of Genes and Genomes. Nucleic Acids Res..

[68] Jin J., Zhang H., Kong L., Gao G., Luo J. (2014). PlantTFDB 3.0: A portal for the functional and evolutionary study of plant transcription factors. Nucleic Acids Res..

[69] Schmittgen T.D., Livak K.J. (2008). Analyzing real-time PCR data by the comparative C(T) method. Nat. Protoc..

[70] Silva F.D., Vasconcelos I.M., Saraiva K.D., Costa J.H., Fernandes C.F., Oliveira J.T. (2019). The expression of the genes involved in redox metabolism and hydrogen peroxide balance is associated with the resistance of cowpea [*Vigna unguiculata* (L.) Walp.] to the hemibiotrophic fungus *Colletotrichum gloeosporioides*. J. Plant Physiol..

