# Supplementary figures and images for: A Comparison of the Transcriptomes of Cowpeas in Response to Two Different Ionizing Radiations
Source: Plants (Basel). 2021 Mar 17;10(3):567. doi: 10.3390/plants10030567 (PMC8002818; doi:10.3390/plants10030567)

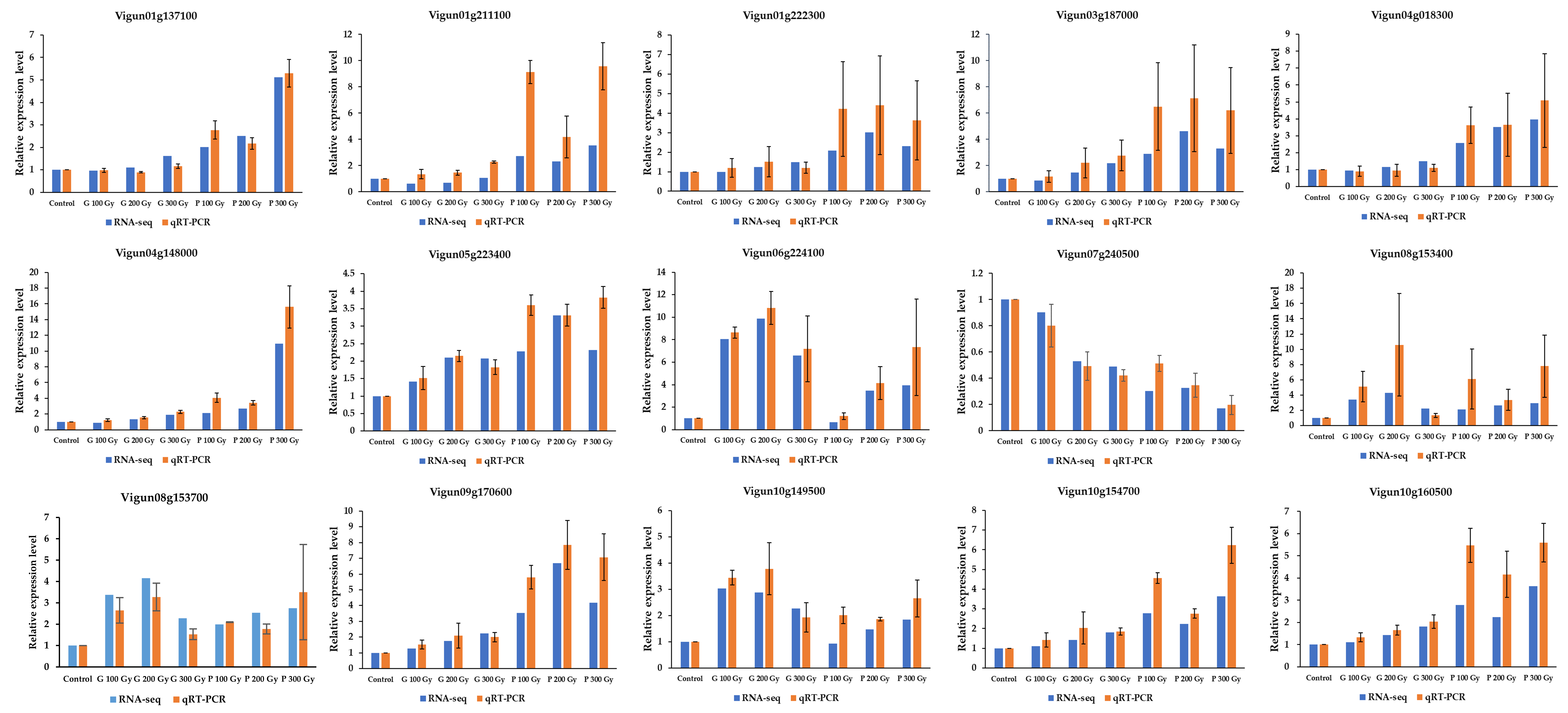

Supplement: Supplementary file 1 [file plants-10-00567-s001.zip › Figure S1. qRT-PCR.tif]
